# Supplementary material for: Tet inactivation disrupts YY1 binding and long-range chromatin interactions during embryonic heart development
Source: Nat Commun. 2019 Sep 20;10:4297. doi: 10.1038/s41467-019-12325-z (PMC6754421; doi:10.1038/s41467-019-12325-z)
Supplement: Supplementary file 1 — Supplementary Information [file 41467_2019_12325_MOESM1_ESM.pdf]

Supplementary Information for

Tet inactivation disrupts YY1 binding and long-range chromatin interactions during embryonic heart development

Fang *et al.*

Figure S1 (related to Figure 1)

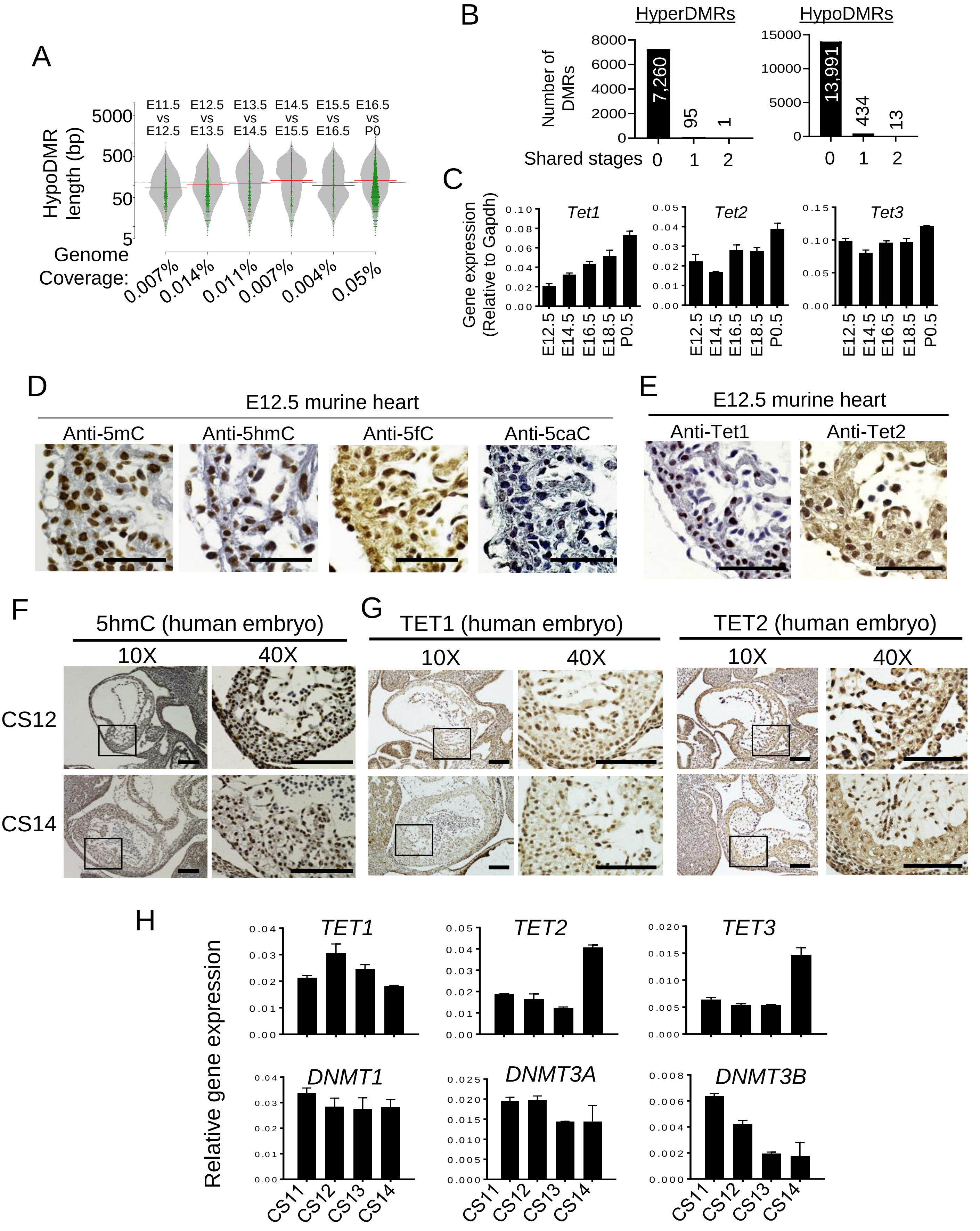

Supplementary Figure 1. Immunohistological analysis on mouse and human embryonic hearts (related to Figure 1).

(A) The average length and genomic coverage of hypoDMR identified from adjacent developmental stages.

(B) The number of common DMRs among different developmental stages. Corrected binomial raw p-value were calculated.

(C)*Tet1, Tet2* and *Tet3* mRNA levels in mouse heart tissues collected at five embryonic stages (E12.5, 14.5, 16.5, 18.5, and P0) determined by quantitative real-time PCR. Data were shown as mean ± S.D; n = 3 independent experiments.

(D) Immunohistochemistry (IHC) analysis on 5mC, 5hmC, 5fC and 5caC distribution patterns in E12.5 mouse embryonic heart tissues. Scale bar: 100 µm.

(E) IHC staining for murine Tet1 and Tet2 in E12.5 mouse embryonic heart tissues.

(F) IHC staining for 5hmC in human embryonic hearts at the developmental stages of CS12 and CS14. Zoomed-in views (40x magnification of boxed regions) were shown next to the corresponding IHC images with 10x magnification.

(G) IHC staining for human TET1 and TET2 in human embryonic hearts at CS12 and CS14 stages.

(H) Expression of *TET* and *DNMT* family members in human embryonic heart tissues (CS11 to CS14) determined by real-time quantitative PCR.

Figure S2 (related to Figure 1)

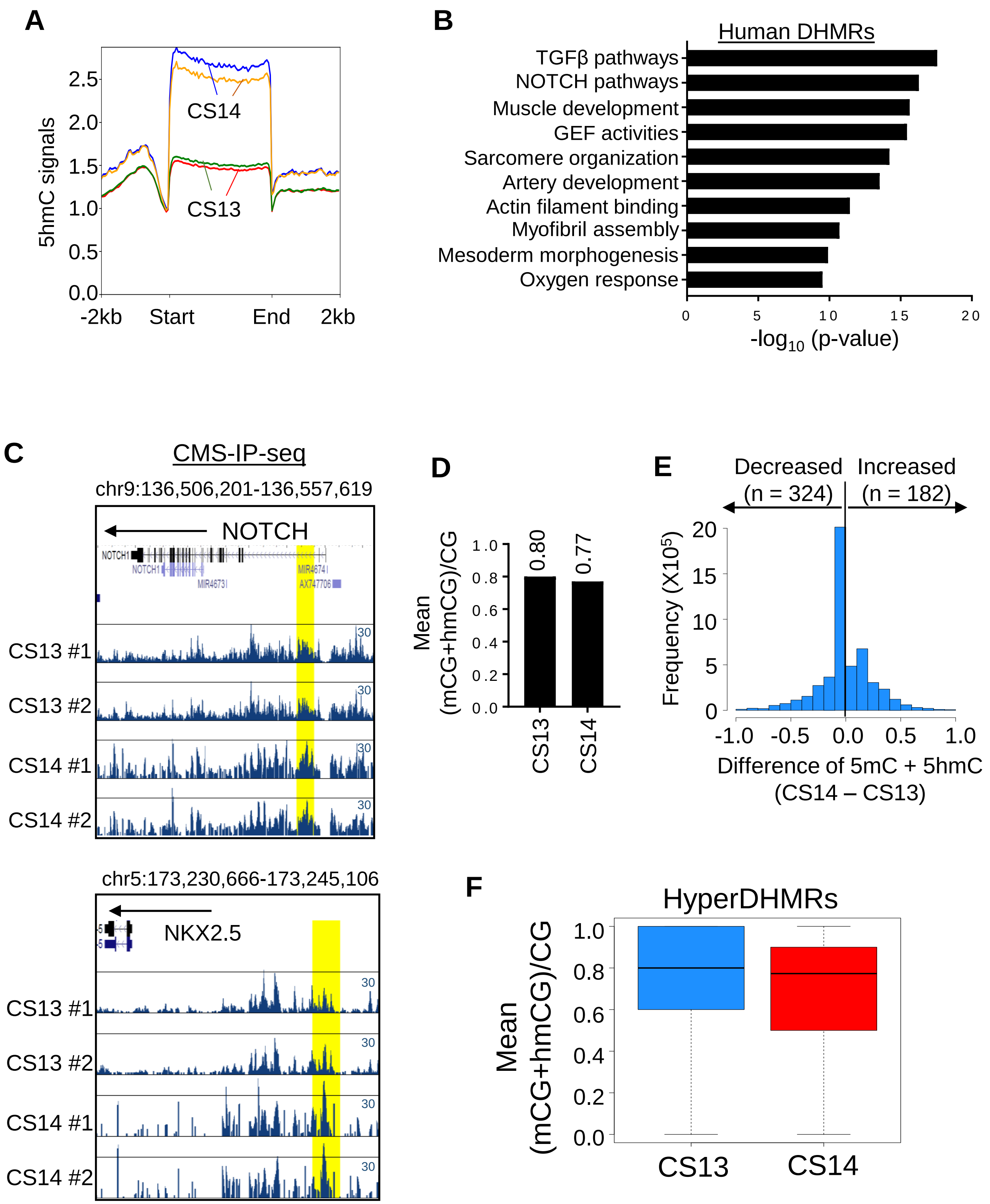

Supplementary Figure 2. DNA methylation and hydroxymethylation dynamics during mouse and human embryonic heart development (related to Figure 1).

(A) Normalized 5hmC enrichment signals over the genebody in human embryonic hearts (CS13, red and green; CS14, blue and yellow).

(B) GREAT analysis on hyperDHMRs from Figure 1H revealed terms associated with embryonic development and heart function.

(C) Representative examples of genome browser views illustrating increased 5hmC signals from CS13 to CS14 located at genomic regions proximal to NOTHC and NKX2.5 loci.

(D) Quantification of global average DNA methylation levels (quantified as mean (mCG+hmCG)/CG) in human heart at CS13 and CS14 based on WGBS analysis.

(E) Comparison of DNA methylation levels between CS14 and CS13 stages measured by WGBS.

(F) Boxplot representation of average DNA methylation levels in hyperDHMRs identified in the human CS13 to CS14 cardiac development stages.

Figure S3 (related to Figure 2)

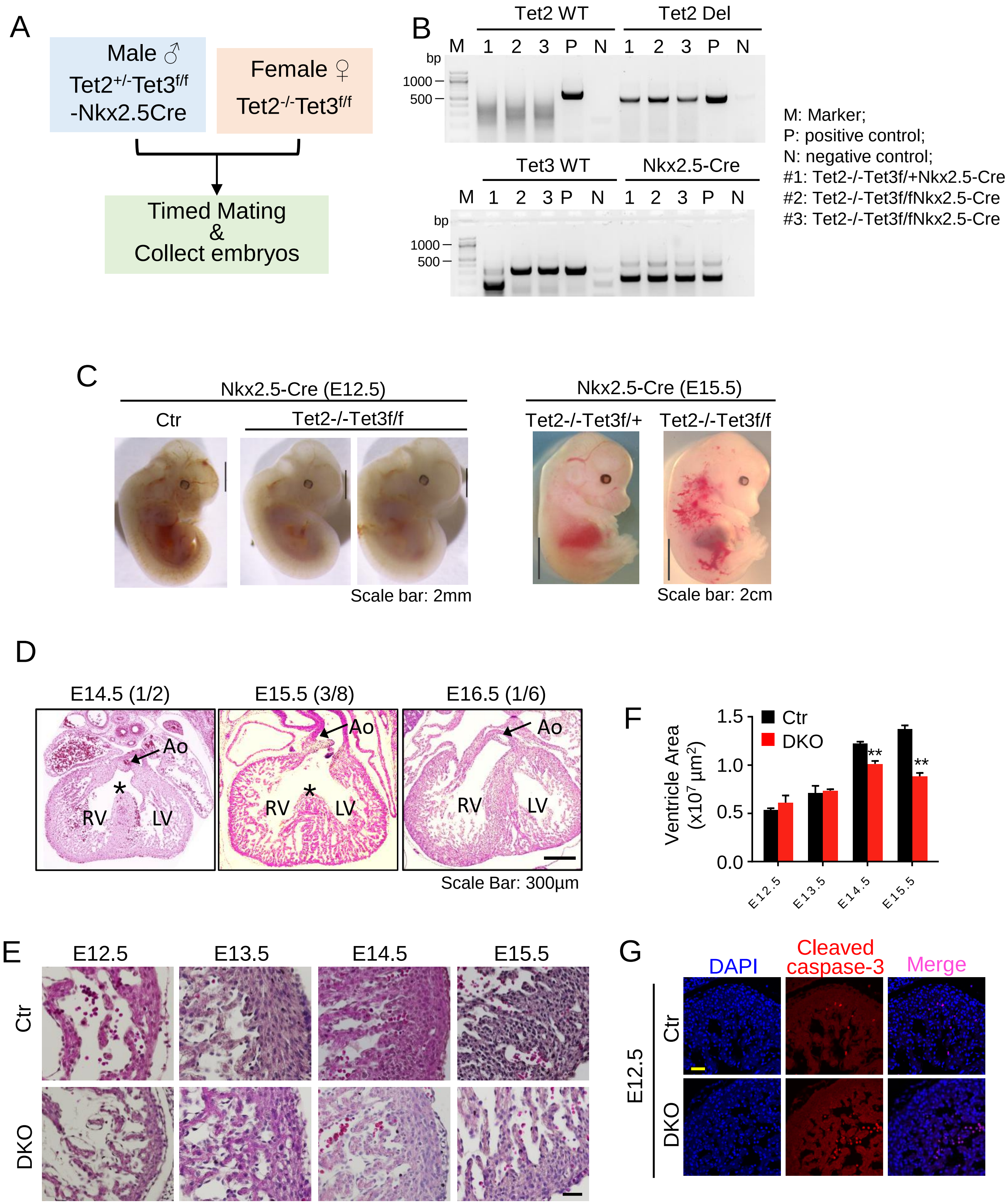

Supplementary Figure 3. Cardiac-specific deletion of Tet2 and Tet3 resulted in developmental defects in the ventricular chambers of embryonic hearts (related to Figure 2).

(A) The breeding strategy to obtain cardiac-specific Tet2/3-DKO embryos.

(B) Representative genotyping results for the detection of genetic alteration of Tet2, Tet3 and Nkx2.5Cre.

(C) Representative images of control and cardiac-specific Tet2/3 modified embryos collected at E12.5 (left) and E15.5 (right).

(D) Representative H&E images of cardiac cross-sections (4X) at E14.5, E15.5, and E16.5 stages. Cardiac developmental defects, including VSD (asterisk) and DORV (arrow), were noted. Shown above the images were numbers of mice displaying developmental defects versus the total amounts of analyzed mice at the corresponding stages. Ao: aorta; RV: right ventricle; LV: left ventricle; DORV: Double outlet right ventricle; VSD: Ventricular septal defects.

(E) Representative H&E staining images of murine heart tissues (40X) collected at E12.5 to 15.5. Scale bar: 500 µm.

(F) Quantifications of the ventricular area in murine heart tissues collected at the indicated developmental stages. ImageJ was used to quantify the ventricular area. Data were shown as mean ± S.D; n = 36 sections from 3 independent experiments. ** $p < 0.01$ when compared to control.

(G) Representative images illustrating IF staining for nuclei (blue; with DAPI staining) and cleaved-caspase 3 (red). Scale bar: 50 µm.

Figure S4 (related to Figure 3)

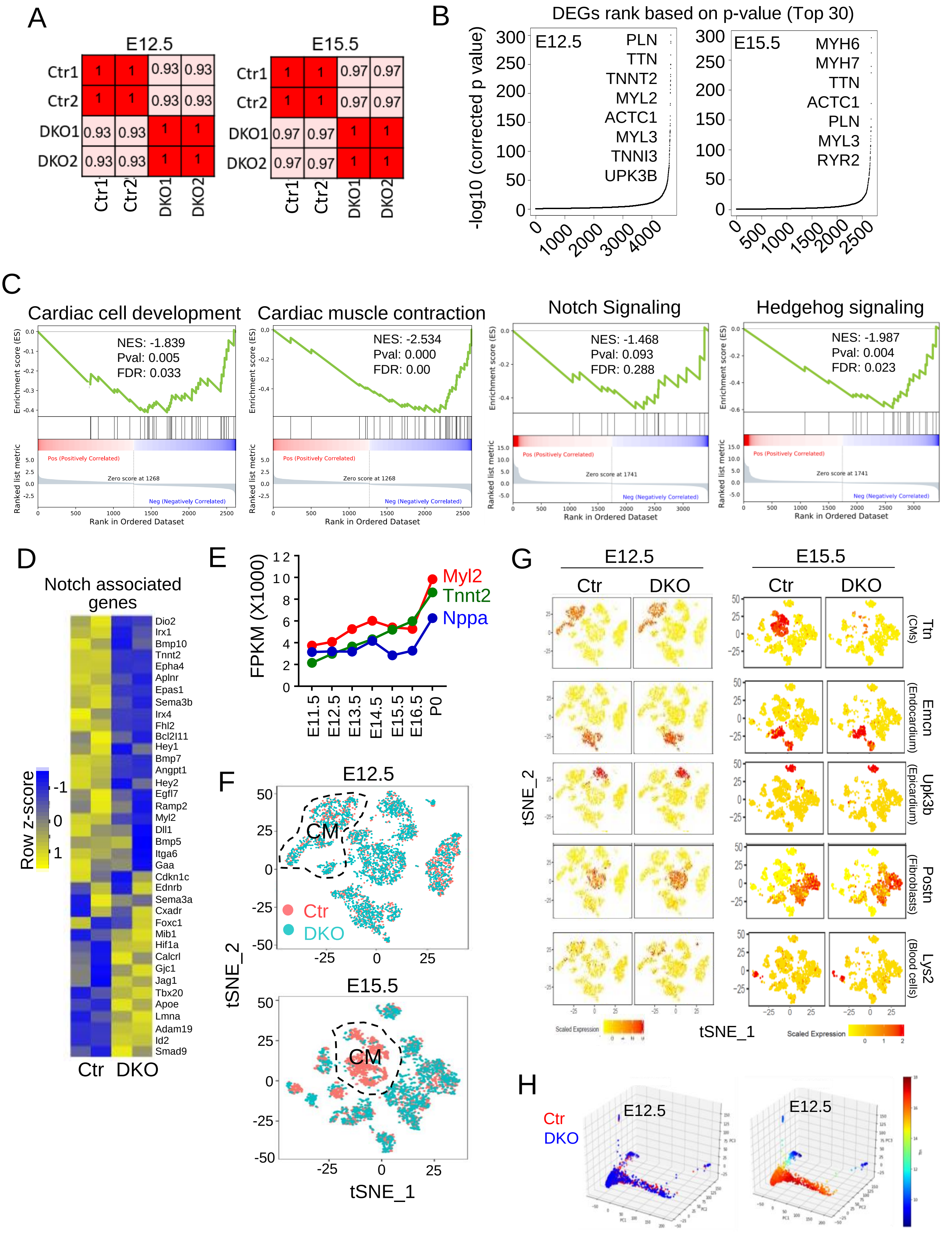

Supplementary Figure 4. Analysis on RNA-seq data obtained from control and Tet2/3-DKO embryonic heart tissues (related to Figure 3).

(A) Summary of Spearman's correlation coefficients between biological replicates of RNA-seq data collected from control and Tet2/3-DKO heart tissues at the E12.5 (left) and E15.5 (right) developmental stages in mice.

(B) Top 30 DEGs were ranked based on their p-values at the E12.5 (left) and E15.5 (right) stages.

(C) GSEA presentation of cardiac-developmental associated terms in identified DEGs. Genes are ranked by fold changes. Hypergeometric test were used to calculate p-value. FDR: Benjamini–Hochberg corrected hypergeometric p-value.

(D) Heatmap representation of the expression levels of genes associated with the Notch signaling pathway associated for the control and Tet2/3-DKO groups (E15.5).

(E) FPKM of selected heart developmental associated genes, *Myl2* (red), *Tnnt2* (green), and *Nppa* (blue), at different developmental stages. RNA-seq data were obtained from ENCODE.

(F) tSNE plot of scRNA-seq data obtained from control (red) and Tet2/3-DKO (cyan) heart tissues at the E12.5 (left) and E15.5 (right) stages.

(G) List of markers used to identify the cell types using scRNA-seq data in E12.5 control and Tet2/3-DKO heart tissue.

(H) 3D PCA plot (left) and *Ttn* expression levels (right) of individual cardiomyocytes based on scRNA-seq data in the control (red) and Tet2/3-DKO (blue) groups at the E12.5 stage.

Figure S5 (related to Figure 4)

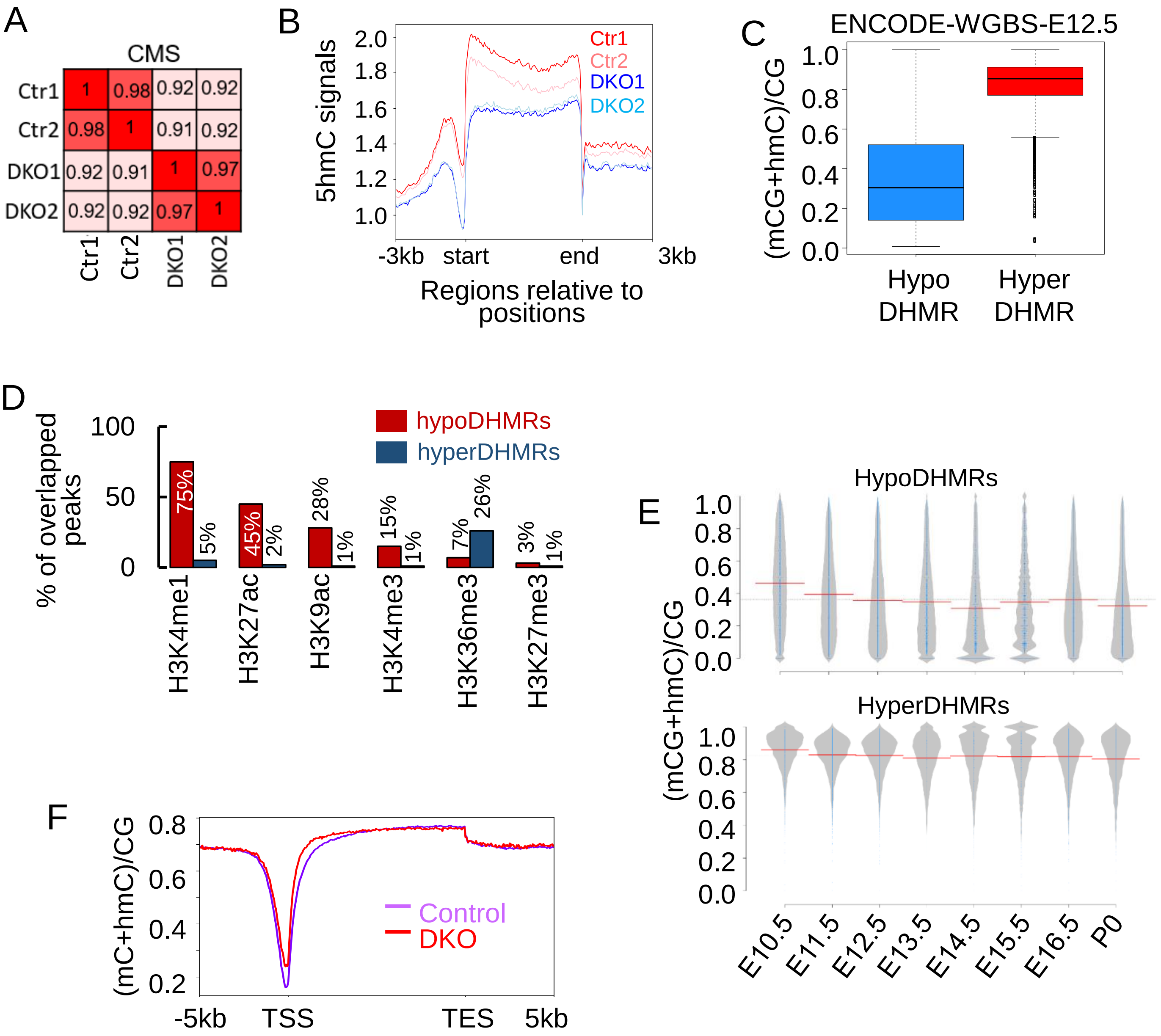

Figure S5 (related to Figure 4)

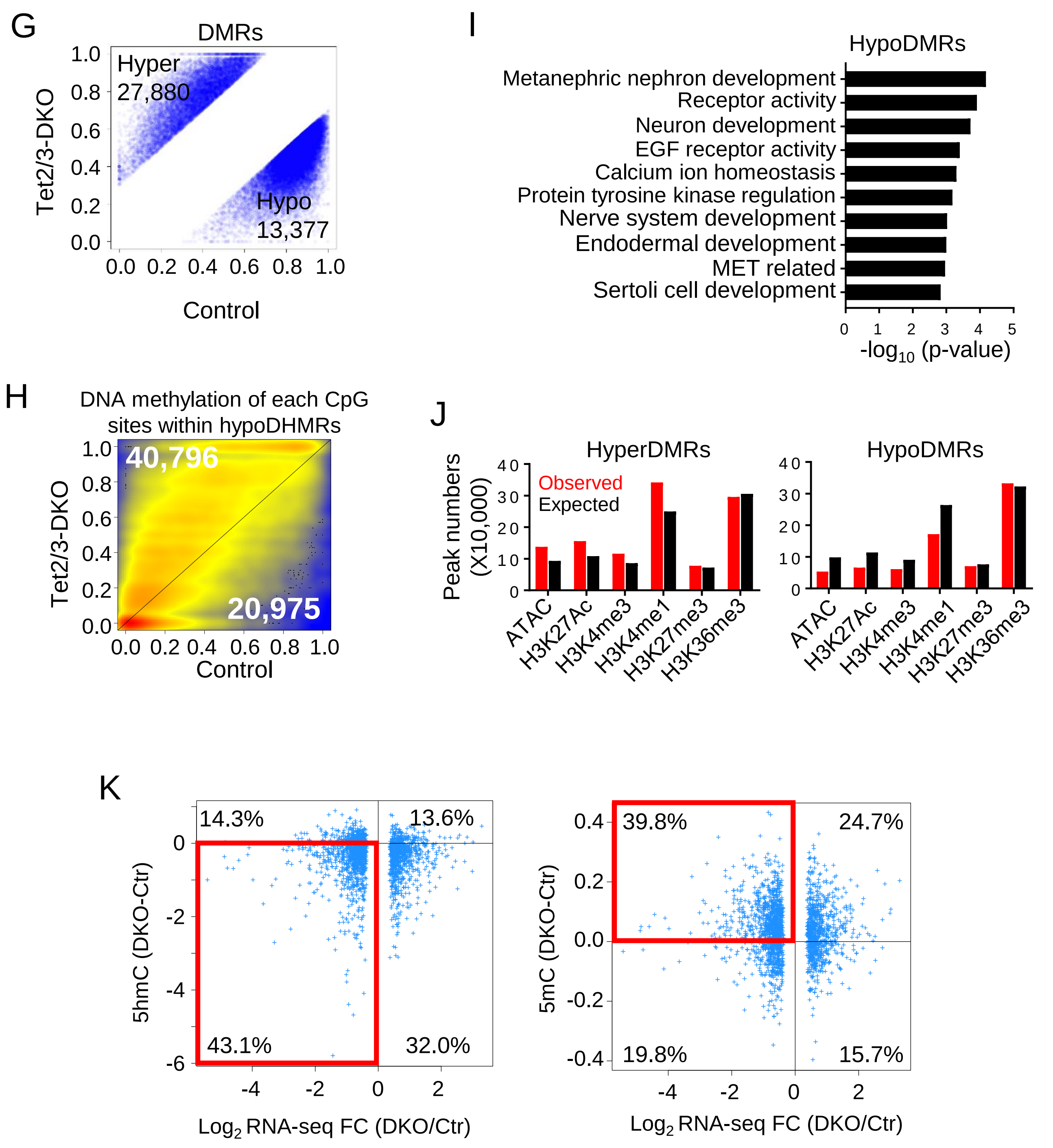

Supplementary Figure 5. Tet2/3 deletion in embryonic hearts impaired 5hmC but not 5mC (related to Figure 4).

(A) Summary of Spearman's correlation coefficients between biological replicates of CMS-IP data collected from control and Tet2/3-DKO E12.5 heart tissues.

(B) Normalized 5hmC enrichment signals over the genebody in mouse embryonic hearts (Red & Pink: control #1 & #2; Blue & Cyan: Tet2/3-DKO #1 & #2).

(C) DNA methylation levels of CpGs within hypoDHMRs and hyperDHMRs identified between the control and Tet2/3-DKO groups. DNA methylation was calculated using WGBS data from E12.5 embryonic hearts deposited in ENCODE.

(D) Percentage of overlapping peaks between hypoDHMRs (red) or hyperDHMRs (blue) and the corresponding histone modifications at E12.5 heart tissues, respectively. The histone modifications data were obtained from the ENCODE database. Total hypoDHMRs and hyperDHMRs are used as denominator to calculate the percentage.

(E) Violin plots showing the distribution and mean value of DNA methylation dynamics at CpGs within hypoDHMR (top) and hyperDHMRs (bottom) identified at the indicated developmental stages (E10.5 to P0). Red line represents the average methylation level. WGBS data were obtained from the ENCODE database.

(F) The distribution profile of average DNA methylation within coding regions in control (purple) and Tet2/3-DKO (red) heart tissues.

(G) Scatterplot of DMRs of in control and Tet2/3-DKO E12.5 heart tissue.

(H) Scatterplot of DNA methylation levels of each CpGs within hypoDHMRs in control and Tet2/3-DKO E12.5 heart tissue.

(I) GREAT analyses on hypoDMRs identified in Tet2/3-DKO heart tissues compared with control. Corrected binomial raw p-value were used.

(J) Observed (red) and expected (black) hyper- (left) or hypo-DMRs (right) that overlapped with corresponding histone marks and chromatin accessible regions measured by ATAC-seq.

(K) Scatterplot depicting the correlation between the change of 5hmC (Y axis) and fold-change in gene expression (X-axis; left panel), and the correlation between the change of DNA methylation levels (Y axis) and fold-change in gene expression alterations (X-axis; right panel) when comparing E12.5 heart tissues from WT and DKO mouse embryos. The red box indicated the genomic regions displaying decreased gene expression with loss of 5hmC or gain of 5mC.

Figure S6 (related to Figure 5)

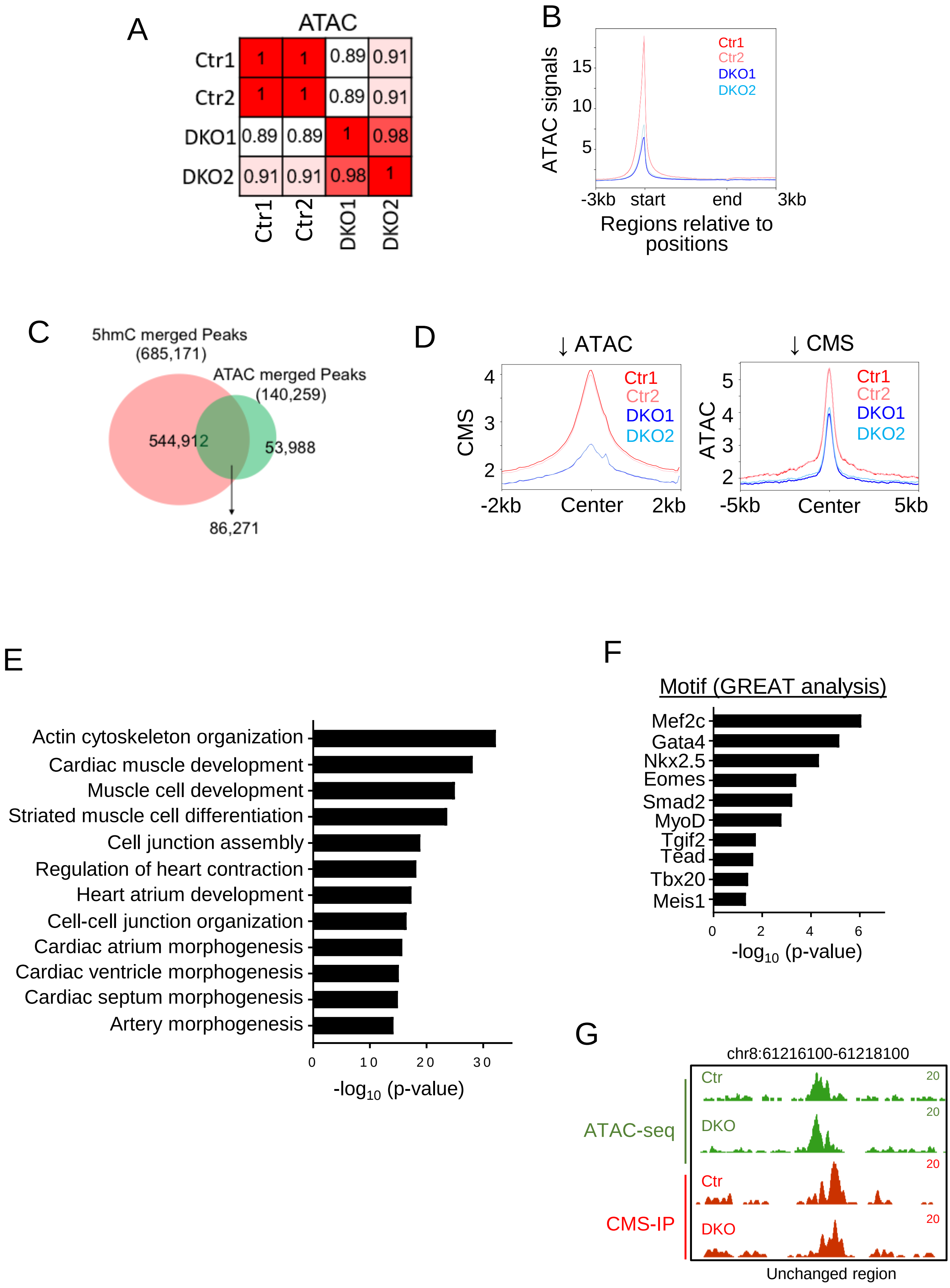

Supplementary Figure 6. Tet2/3 deletion in embryonic hearts compromised chromatin accessibility and YY1 binding to the chromatin (related to Figure 5).

(A) Summary of Spearman's correlation coefficients between biological replicates of ATAC-seq data collected from WT and Tet2/3-DKO E12.5 heart tissues.

(B) Normalized ATAC-seq signals over the genebody in mouse embryonic hearts (Red & Pink: control 1 & 2; Blue & Cyan: Tet2/3-DKO1 & 2).

(C) Venn diagram showing the degree of overlaps between 5hmC and ATAC-seq enriched genomic regions.

(D) (Left) Enrichment of 5hmC signals (left) at genomic regions displaying reduced ATAC-seq signals in the Tet2/3-DKO group compared to control. (Right) Enrichment of ATAC-seq signals at genomic regions displaying decreased 5hmC enrichment in the Tet2/3-DKO group compared to WT. Red & Pink: control 1 & 2; Blue & Cyan: Tet2/3-DKO1 & 2.

(E) GREAT analysis of genomic regions showing reduction in both 5hmC and ATAC-seq signals in the Tet2/3-DKO group compared to control. Corrected binomial raw p-value were used.

(F) Motif analysis of genomic regions displaying decreased 5hmC and ATAC-seq signals in the Tet2/3-DKO group. Benjamini–Hochberg corrected hypergeometric were used to calculate p-value

(G) Genome browser views of genomic regions that showed no change of chromatin accessibility (top, green) and 5hmC enrichment (red, bottom) in WT and DKO embryonic heart tissues collected at the E12.5 stage.

Figure S7 (related to Figure 6)

A

```
Tet1     5′ TTCCTCACCTAGTCTCCATGAGCTCCCTGACAGCAGCCACACT 3′
Clone 1     TTCCTCACCTAGTCT-------------GACAGCAGCCACACT  -13bp
Clone 2     TTCCTCACCTAGTCTCCA---------TGACAGCAGCCACACT  -9bp

Tet2     5′ AAACACGTGAAAGTGCCAACAGATATCCAGGCTGCAGAATCGG 3′
Clone 1     AAACACGTGAAAGTGCCAACAG--ATCCAGGCTGCAGAATCGG  -2bp
Clone 2     AAACACGTGAAAGTGCCAACAGAT-------CTGCAGAATCGG  -7bp

Tet3     5′ CCACTTGGCGATGGGACAGCCTCGAGAACTCTTCCCCTCCTTG 3′
Clone 1     CCACTTGGCGATGGGACAGC--------CTCTTCCCCTCCTTG 3′ -8bp
Clone 2     CCACTTGGCGATGGGACAG----------------CCTCCTTG 3′ -16bp
```

B

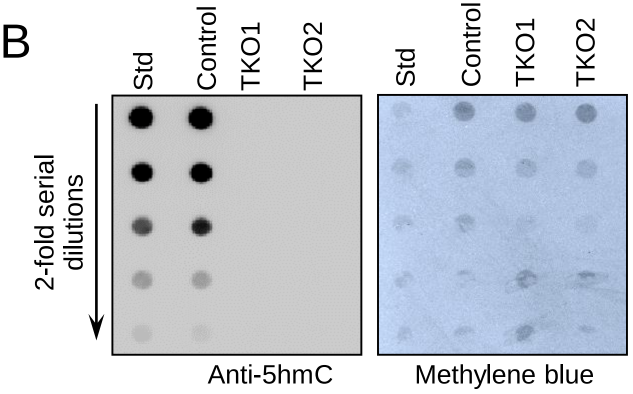

C

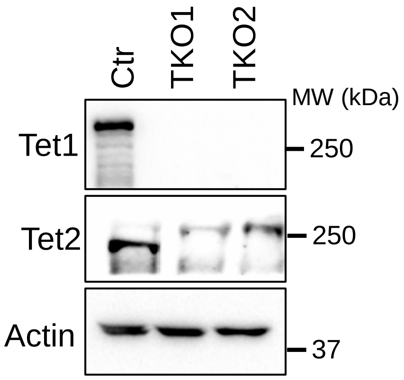

Supplementary Figure 7. Generating Tet1/2/3 triple knockout mESC (related to Figure 6)

(A) Amplicon sequencing of CRISPR/Cas9 targeted regions of Tet1, 2, 3 to confirm the gene disruption in mESCs.

(B) Dot-blot analysis of global 5hmC levels in WT and Tet-TKO mESCs.

(C) Western blotting analysis of Tet1 and Tet2 protein level in WT and Tet-TKO mESCs.

Figure S8 (related to Figure 6)

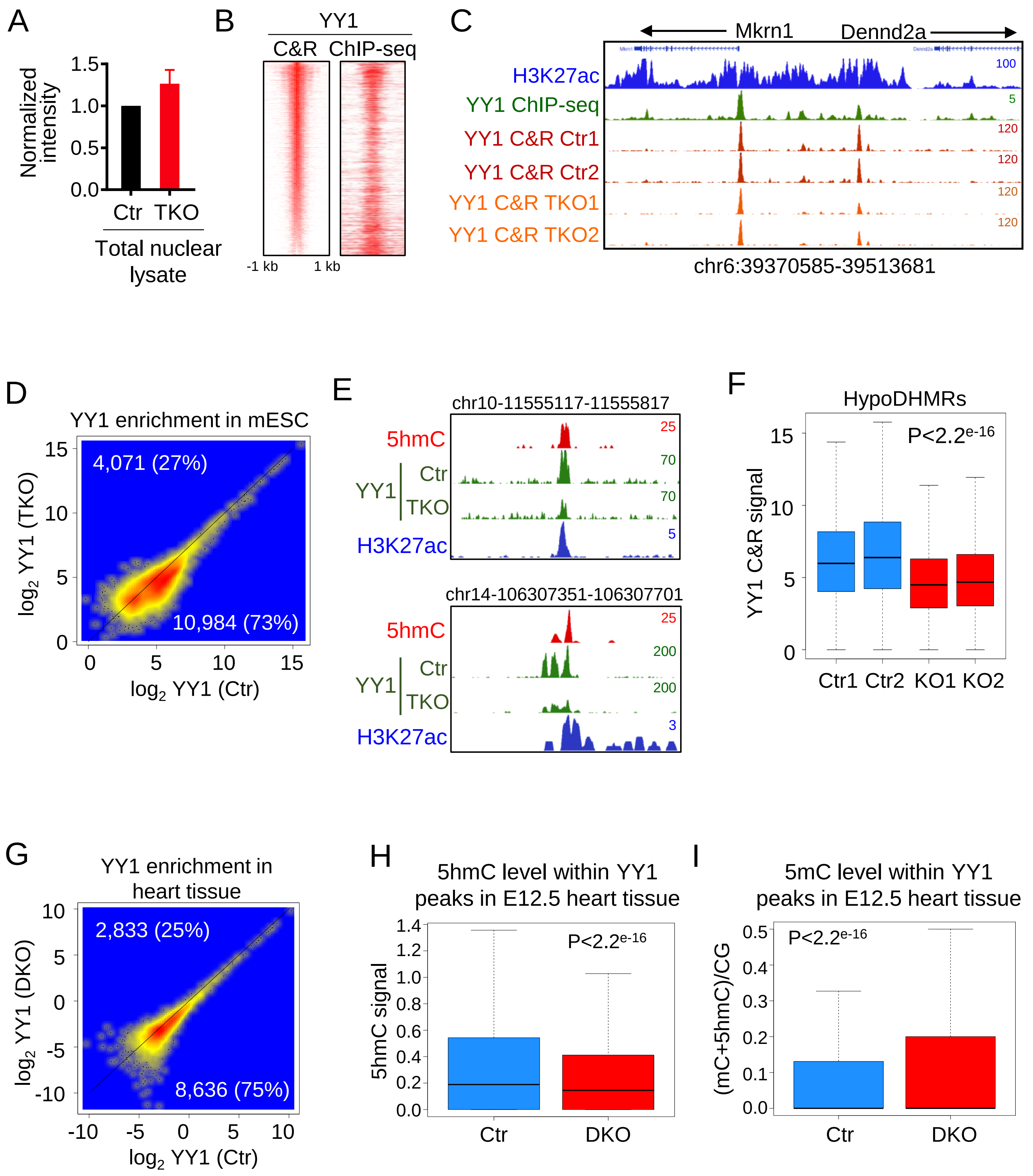

Supplementary Figure 8. Tet triple knockout compromised YY1 binding to its genomic targets in mESCs (related to Figure 6).

(A) Quantification of the intensity of the total amount of YY1 in nuclear lysate from WT and Tet-TKO mESCs as shown in Figure 5F. Data were shown as mean ± S.D; n = 3 independent experiments.

(B) Heatmap representation of YY1 enriched regions using the CUT&RUN and published ChIP-seq dataset (GSM1665561) in mESCs.

(C) Genome browser views showing the comparison between the CUT&RUN (red & orange) and published ChIP-seq (green) methods to capture YY1 enriched regions at the Mkm1 and Dennd2a loci in mESCs.

(D) Scatterplot depicting the YY1 enrichment in WT (X-axis) and Tet-TKO (Y-axis) mESCs.

(E) Genome browser views of genomic regions that showed 5hmC enrichment (red), YY1 binding (green) and H3K27ac enrichment (blue) in mESCs.

(F) YY1 enrichment within hypoDHMRs identified from WT and Tet-TKO mESCs. Kolmogorov–Smirnov test were used to calculate p-value.

(G) Scatterplot depicting the YY1 enrichment in the control (X-axis) and Tet-DKO (Y-axis) heart tissues collected at the E12.5 stage.

(H) 5hmC enrichment within YY1 peaks in the control (blue) and DKO (red) E12.5 embryonic heart tissues. Kolmogorov–Smirnov test were used to calculate p-value.

(I) Quantification of 5mC and 5hmC levels within YY1 peaks in the control (blue) and DKO (red) embryonic heart tissues at the E12.5 stage. Kolmogorov–Smirnov test were used to calculate p-value.

Figure S9 (related to Figure 7)

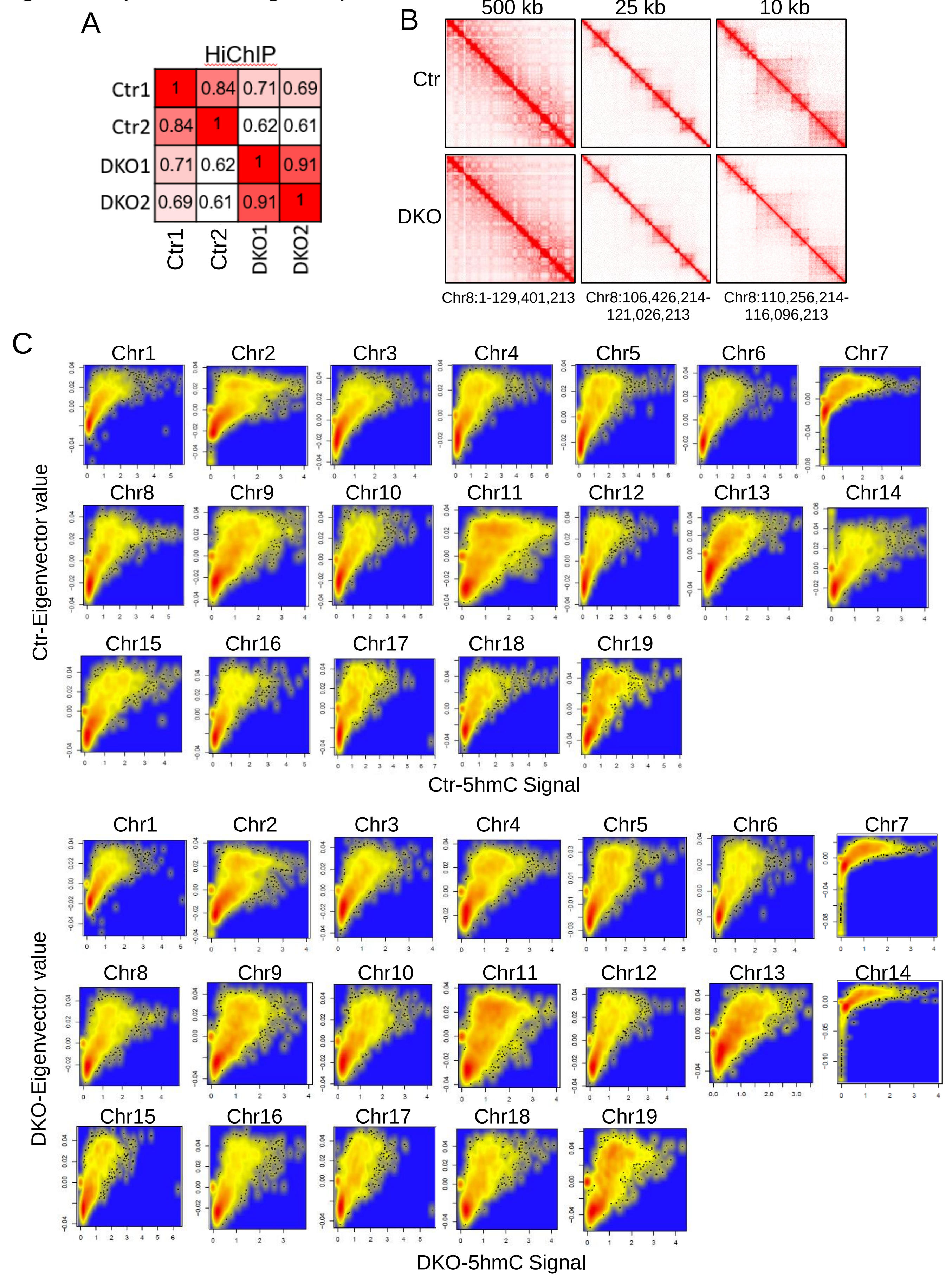

Figure S9 (related to Figure 7)

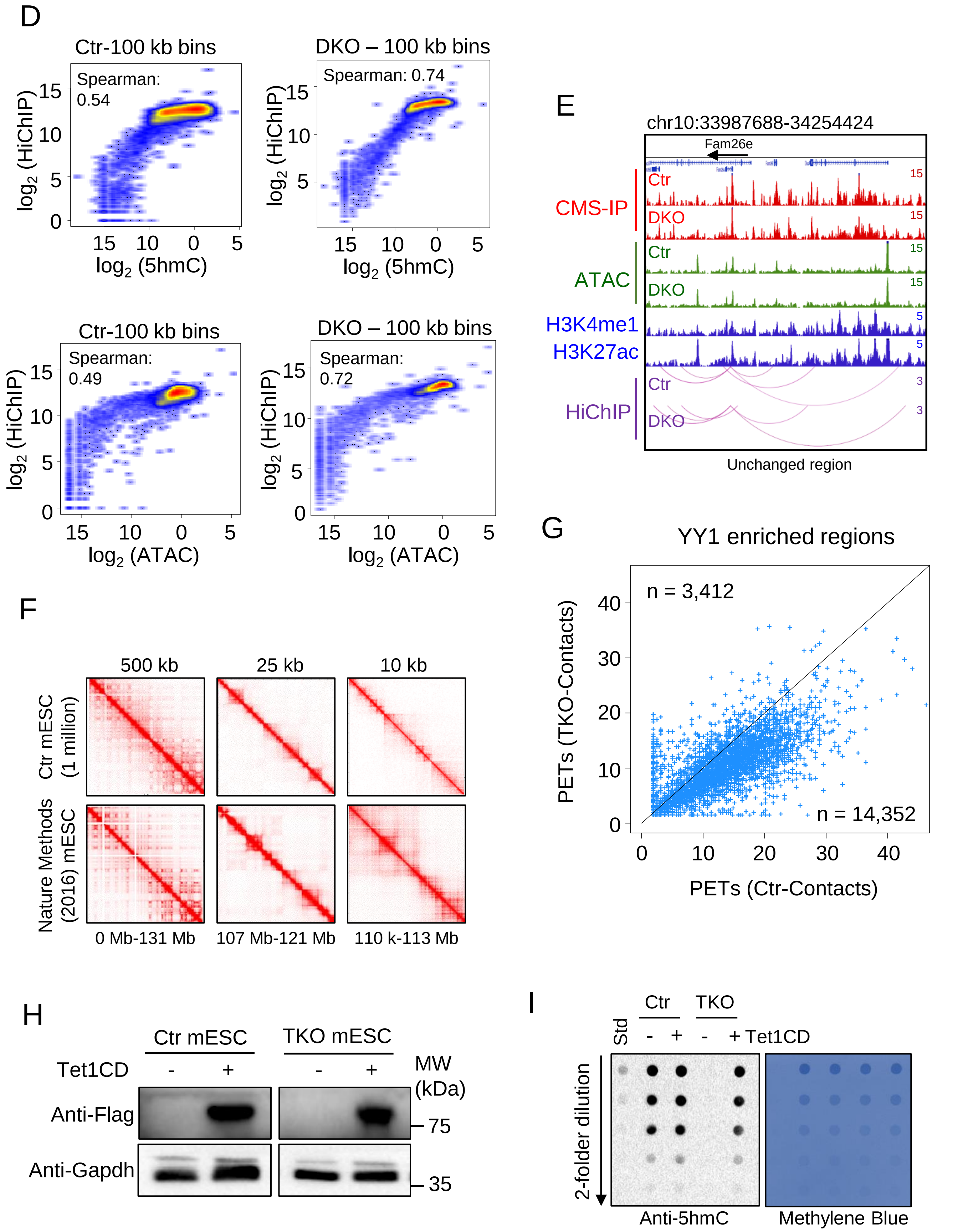

Supplementary Figure 9. Tet-mediated DNA hydroxymethylation is associated with chromatin higher-order structures (related to Figure 6).

(A) Summary of Spearman's correlation coefficients between biological replicates of Hi-ChIP data collected in control and Tet2/3-DKO E12.5 heart tissues.

(B) Heatmap representation of chromatin interactions in control and Tet2/3-DKO heart tissues (E12.5) at 500kb, 25kb, and 10kb resolution within chromosome 8.

(C) Scatterplots showing the correlation between 5hmC signals and Eigenvector value calculated at each chromosome in control (top) and Tet2/3-DKO (bottom) heart tissue (E12.5). Eigenvector and 5hmC signals in every 50 k bins along the whole genome were calculated, respectively.

(D) Scatterplots showing the calculated correlation between 5hmC (top) or ATAC-seq (bottom) signals and HiChIP signals in the control and Tet2/3-DKO heart tissues (E12.5).

(E) Representative genome browser views of genomic regions displaying no changes of 5hmC (red), chromatin accessibility (green), and promoter-enhancer looping (purple) in the control and DKO E12.5 mouse heart tissues.

(F) Heatmap representation of chromatin interactions in WT and Tet-TKO mESCs at 500kb, 25kb, and 10kb resolution within chromosome 8.

(G) Scatterplot representation of PETs in WT and Tet-TKO mESCs within the YY1 enriched regions.

(H) Immunoblot analysis of Flag-Tet1CD expression in WT and Tet-TKO mESCs.

(I) 5hmC dot-blot analysis in WT and Tet-TKO mESCs with and without the expression of FLAG-Tet1CD (left). Methylene blue staining on the same blot was used as the total DNA loading control (right).
