# Supplementary material for: Tet inactivation disrupts YY1 binding and long-range chromatin interactions during embryonic heart development
Source: Nat Commun. 2019 Sep 20;10:4297. doi: 10.1038/s41467-019-12325-z (PMC6754421; doi:10.1038/s41467-019-12325-z)
Supplement: Supplementary file 3 — Description of Additional Supplementary Files [file 41467_2019_12325_MOESM3_ESM.pdf]

Description of Additional Supplementary Files

File Name: Supplementary Data 1
Description: NGS data information.

File Name: Supplementary Data 2
Description: List of primers and oligos used in this manuscript.

File Name: Supplementary Data 3
Description: Single cell RNA seq gene expression profile in each cluster.
