# Supplementary material for: Tet inactivation disrupts YY1 binding and long-range chromatin interactions during embryonic heart development
Source: Nat Commun. 2019 Sep 20;10:4297. doi: 10.1038/s41467-019-12325-z (PMC6754421; doi:10.1038/s41467-019-12325-z)
Supplement: Supplementary file 7 — Reporting Summary [file 41467_2019_12325_MOESM7_ESM.pdf]

nature research

Corresponding author(s): Deqiang Sun
James F. Martin
Yun Huang

Last updated by author(s): Jul 29, 2019

Reporting Summary

Nature Research wishes to improve the reproducibility of the work that we publish. This form provides structure for consistency and transparency in reporting. For further information on Nature Research policies, see Authors & Referees and the Editorial Policy Checklist.

Statistics

For all statistical analyses, confirm that the following items are present in the figure legend, table legend, main text, or Methods section.

n/a	Confirmed	
☐	☒	The exact sample size (*n*) for each experimental group/condition, given as a discrete number and unit of measurement
☐	☒	A statement on whether measurements were taken from distinct samples or whether the same sample was measured repeatedly
☐	☒	The statistical test(s) used AND whether they are one- or two-sided<br>*Only common tests should be described solely by name; describe more complex techniques in the Methods section.*
☒	☐	A description of all covariates tested
☒	☐	A description of any assumptions or corrections, such as tests of normality and adjustment for multiple comparisons
☐	☒	A full description of the statistical parameters including central tendency (e.g. means) or other basic estimates (e.g. regression coefficient) AND variation (e.g. standard deviation) or associated estimates of uncertainty (e.g. confidence intervals)
☐	☒	For null hypothesis testing, the test statistic (e.g. *F*, *t*, *r*) with confidence intervals, effect sizes, degrees of freedom and *P* value noted<br>*Give P values as exact values whenever suitable.*
☐	☒	For Bayesian analysis, information on the choice of priors and Markov chain Monte Carlo settings
☐	☒	For hierarchical and complex designs, identification of the appropriate level for tests and full reporting of outcomes
☐	☒	Estimates of effect sizes (e.g. Cohen's *d*, Pearson's *r*), indicating how they were calculated

Our web collection on statistics for biologists contains articles on many of the points above.

Software and code

Policy information about availability of computer code

Data collection	Dissecting microscopy (Nikon SMZ800N)<br>Eclipse Ci microscopy (Nikon)<br>Confocal microscopy (Nikon A1)<br>ChemiDoc imaging system (Bio-Rad)<br>LightCycle 96 (Roche)
Data analysis	Image/Western blot analysis: Fiji (Image J)<br>Statistical analysis: Excel 2015<br>Bulk RNA-Seq data analysis: tophat/2.1.1; GSEA(Broad Institute); Rstudio/1.2.1335<br>Single Cell RNA-seq data analysis: CellRanger/V2.1.1 (10XGenomics); R package Seruat/V2.3 (Satjia lab); MAGIC(KrishnaswamyLab)<br>WGBS and CMSIP analysis: bsmap/2.89; MOABS (Li lab); GREAT (Bejerano lab); DEGseq2 (Bioconductor); Mmint<br>ATAC-Seq analysis: Bowtie2; MACS2; Bedtools; bigwigOverbed; Homer/v4.10.5<br>HiChIP analysis: HiC-Pro; Fit-Hi-C; WashU Epigenome Browser<br>4C-seq analysis: Basic4seq (R package)

For manuscripts utilizing custom algorithms or software that are central to the research but not yet described in published literature, software must be made available to editors/reviewers. We strongly encourage code deposition in a community repository (e.g. GitHub). See the Nature Research guidelines for submitting code & software for further information.

Data

Policy information about availability of data

All manuscripts must include a data availability statement. This statement should provide the following information, where applicable:

- Accession codes, unique identifiers, or web links for publicly available datasets
- A list of figures that have associated raw data
- A description of any restrictions on data availability

DNA methylation and Histone modifications (H3K27ac/H3K4me1/H3K4me3/H3K9ac/H3K27me3/H3K36me3/H3K9me3) from different embryonic mouse heart developmental stages were analyzed by using ECONDE data. YY1 ChIP-Seq data(GSM1665561) for E14 WT mESCs was used to compare with our YY1 cut&run data.
All of the raw sequencing data for this work are under the GEO accession number GSE121671 and it is currently not public available. Figures associated with these raw data are Figure1, Figure3, Figure4, Figure5, Figure6, Figure7, Figure S2, Figure S4, Figure S5, Figure S6, Figure S8 and Figure S9. Genome browser visualization of the raw data is public available from this track: http://epigenomegateway.wustl.edu/browser/?genome=mm10&session=HxzfOrp6Ca&statusId=402691614

Field-specific reporting

Please select the one below that is the best fit for your research. If you are not sure, read the appropriate sections before making your selection.

☒ Life sciences ☐ Behavioural & social sciences ☐ Ecological, evolutionary & environmental sciences

For a reference copy of the document with all sections, see nature.com/documents/nr-reporting-summary-flat.pdf

Life sciences study design

All studies must disclose on these points even when the disclosure is negative.

Sample size	Sample size was decided by our experience with the techniques in this research. From our statistic results, we are confident that the sample size we used was sufficient to support the differences between control and knock-out groups.
Data exclusions	None
Replication	Biological replicates (>=2) were performed for bulk RNA-seq , WGBS, CMSIP, ATAC-seq, HiChIP and 4C-seq experiments. We also performed biological replicates for the western blot, dot blot and qPCR experiments by collecting materials from independent samples. The replicates number is reported in the figure legend.
Randomization	There was no randomization in this study. We analyzed the embryos with different genotypes at different developmental stages. We ensured that control and knock-out embryos were at similar developmental stages by counting the somite pairs.
Blinding	Investigators were not blinded in this study

Reporting for specific materials, systems and methods

We require information from authors about some types of materials, experimental systems and methods used in many studies. Here, indicate whether each material, system or method listed is relevant to your study. If you are not sure if a list item applies to your research, read the appropriate section before selecting a response.

Materials & experimental systems

n/a	Involved in the study	
☐	☒	Antibodies
☐	☒	Eukaryotic cell lines
☒	☐	Palaeontology
☐	☒	Animals and other organisms
☒	☐	Human research participants
☒	☐	Clinical data

Methods

n/a	Involved in the study	
☒	☐	ChIP-seq
☒	☐	Flow cytometry
☒	☐	MRI-based neuroimaging

Antibodies

Antibodies used	Tet1 antibody, kindly provided by Dr. Leonhardt Heinrich<br>anti-Tet2, Abcam, ab124297<br>anti-5mC, Millipore, MABE146<br>anti-5hmC, Active Motif, 39769<br>anti-5fC, Active Motif, 61223<br>anti-5caC, Active Motif, 61225

	anti-Ki67, Abcam, ab16667<br>anti-cleaved caspase-3, Cell Signaling Technologies, 9661s<br>Alexa Fluor 568 goat anti-rabbit, Thermo Fisher Scientific, A-11011<br>Alexa Fluor 647 goat anti-mouse, Thermo Fisher Scientific, A-21235<br>anti-YY1, Santa Cruz, sc-7341<br>anti-H3K27ac, Abcam, ab4729<br>Rabbit anti-mouse, Abcam, ab6728<br>anti-cTNT, Thermal Fisher Scientific, 13-11<br>anti-Smc1, Bethyl Laboratories, Inc., A300-055A,<br>anti-YY1, Santa Cruz, sc-7341<br>anti-H3, Abcam, ab1791<br>anti-Flag, Sigma, M2
Validation	Tet1 antibody, validated by IHC<br>anti-Tet2, validated by IHC<br>anti-5mC, validated by IHC<br>anti-5hmC, validated by IHC, IF and dot blot<br>anti-5fC, validated by IHC<br>anti-5caC, validated by IHC<br>anti-Ki67, validated by IF<br>anti-cleaved caspase-3, validated by IF<br>Alexa Fluor 568 goat anti-rabbit, validated by IF<br>Alexa Fluor 647 goat anti-mouse, validated by IF<br>anti-YY1, validated by western blot and Cut&Run<br>anti-H3K27ac, validated by Cut&Run<br>Rabbit anti-mouse, validated by Cut&Run<br>anti-cTNT, IF<br>anti-Smc1, validated by HiChIP<br>anti-H3, validated by Western blot<br>anti-Flag, validated by Western blot

Eukaryotic cell lines

Policy information about cell lines

Cell line source(s)	HEK 293T Cells<br>E14 Nkx2.5-GFP mESCs
Authentication	Authenticated
Mycoplasma contamination	cell are mycoplasma negative
Commonly misidentified lines<br>(See ICLAC register)	none

Animals and other organisms

Policy information about studies involving animals; ARRIVE guidelines recommended for reporting animal research

Laboratory animals	C57BL/6, Nkx2.5-Cre, male, age 2-4 months old<br>C57BL/6, Tet2+/-Tet3flox/flox;Nkx2.5-Cre, male, age 2-4 months<br>C57BL/6, WT, female, age 2-4 months<br>C57BL/6, Tet2-/-Tet3flox/flox, female, age 2-4 months
Wild animals	not involved in this study
Field-collected samples	not involved in this study
Ethics oversight	Animal studies were approved by the Institutional Animal Care Use Committee (IACUC) of the Institute of Biosciences and Technology, Texas A&M University.

Note that full information on the approval of the study protocol must also be provided in the manuscript.
