# Supplementary figures and images for: Tet inactivation disrupts YY1 binding and long-range chromatin interactions during embryonic heart development
Source: Nat Commun. 2019 Sep 20;10:4297. doi: 10.1038/s41467-019-12325-z (PMC6754421; doi:10.1038/s41467-019-12325-z)

Figure 1E

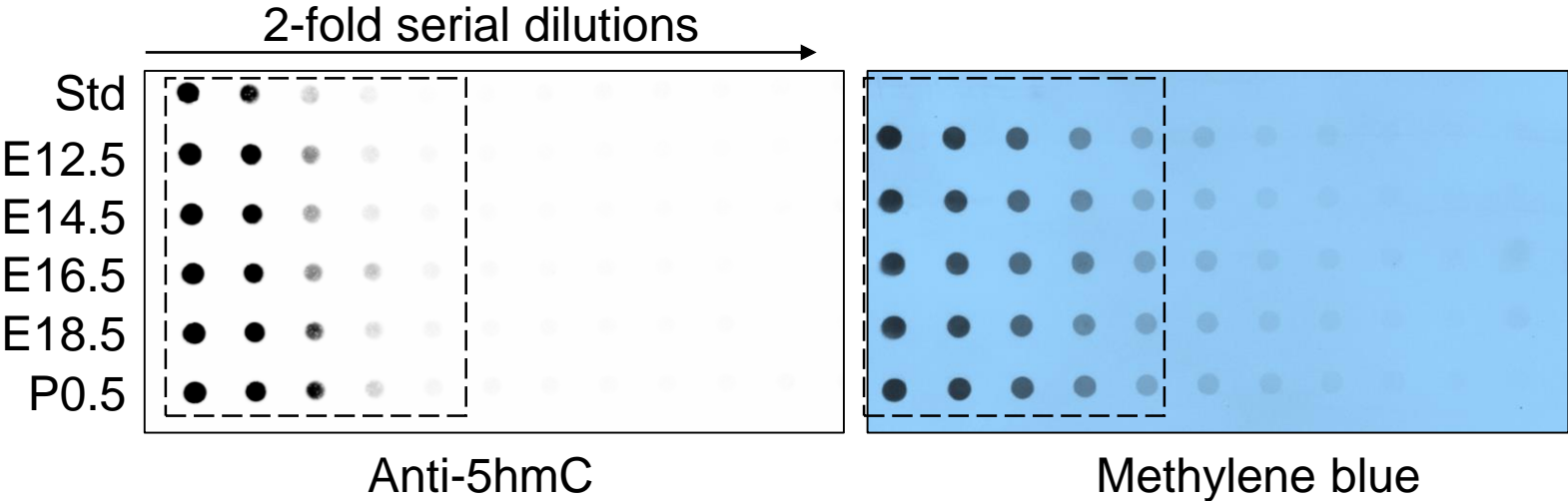

Figure 4B

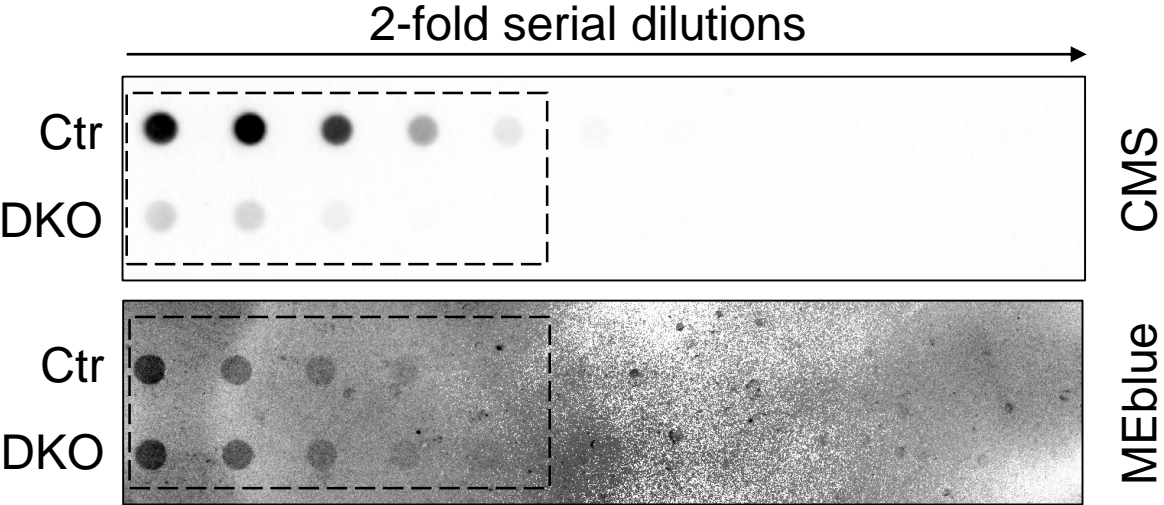

Figure 6A

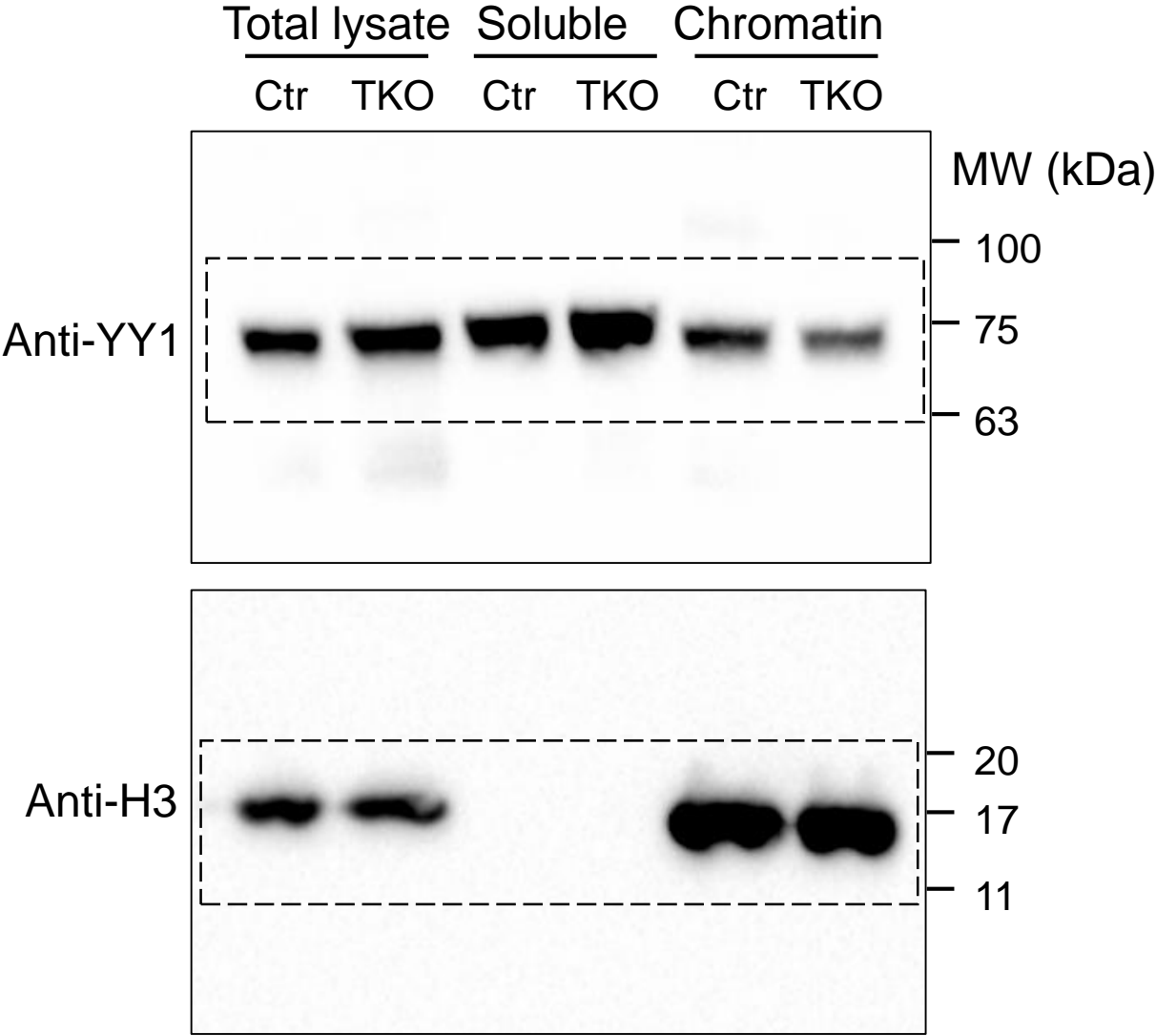

Figure S3B

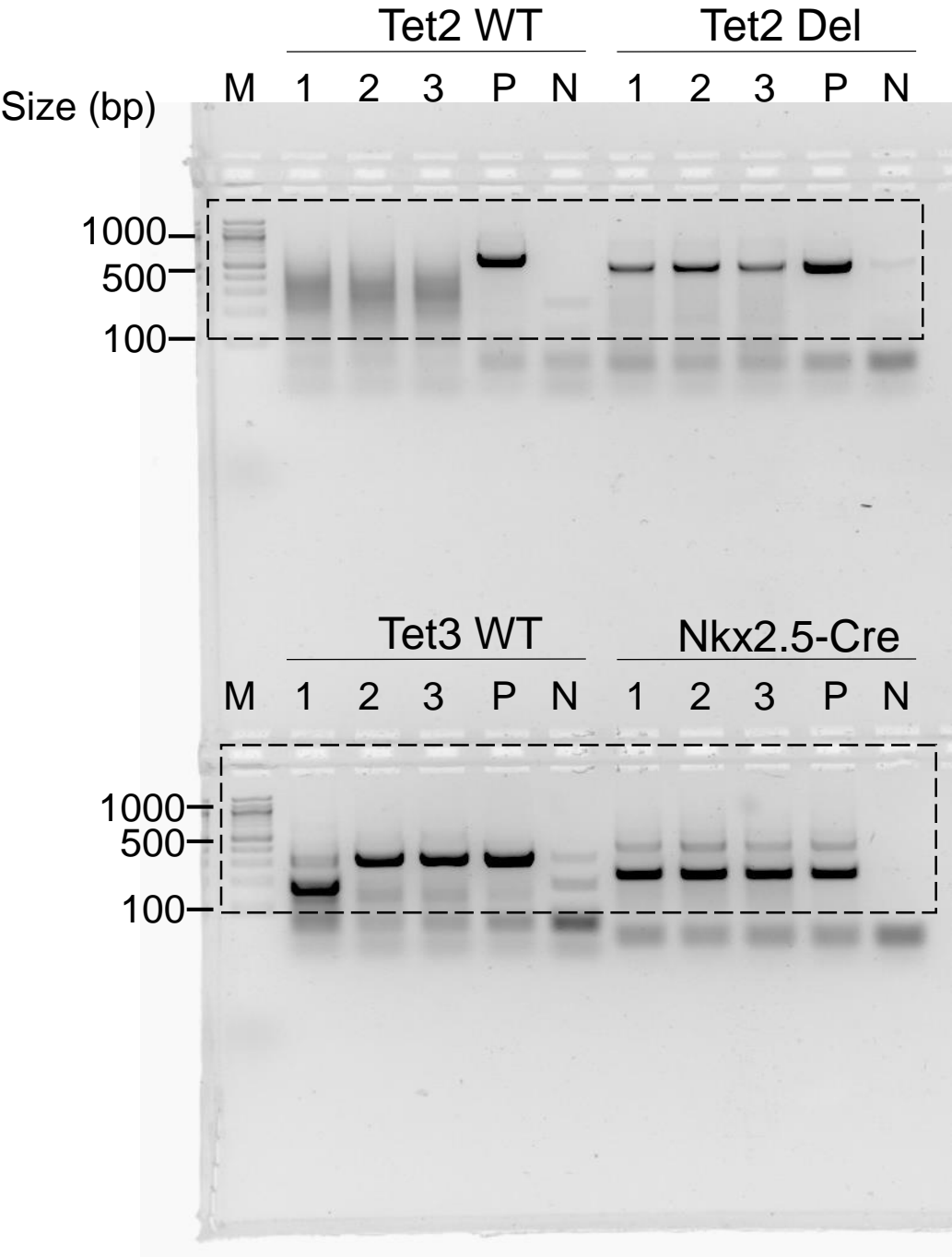

Figure S7B

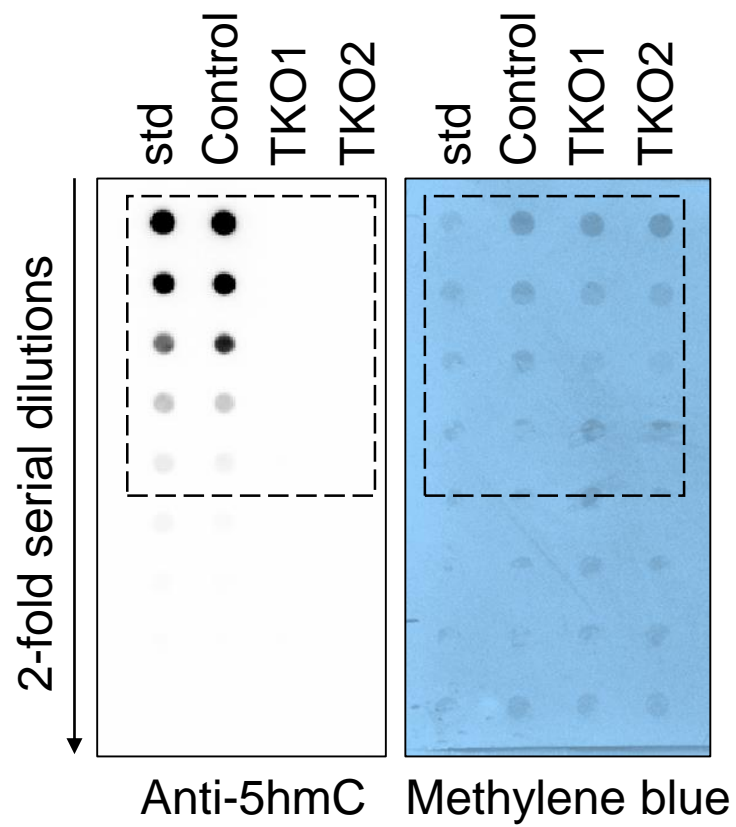

Figure S7C

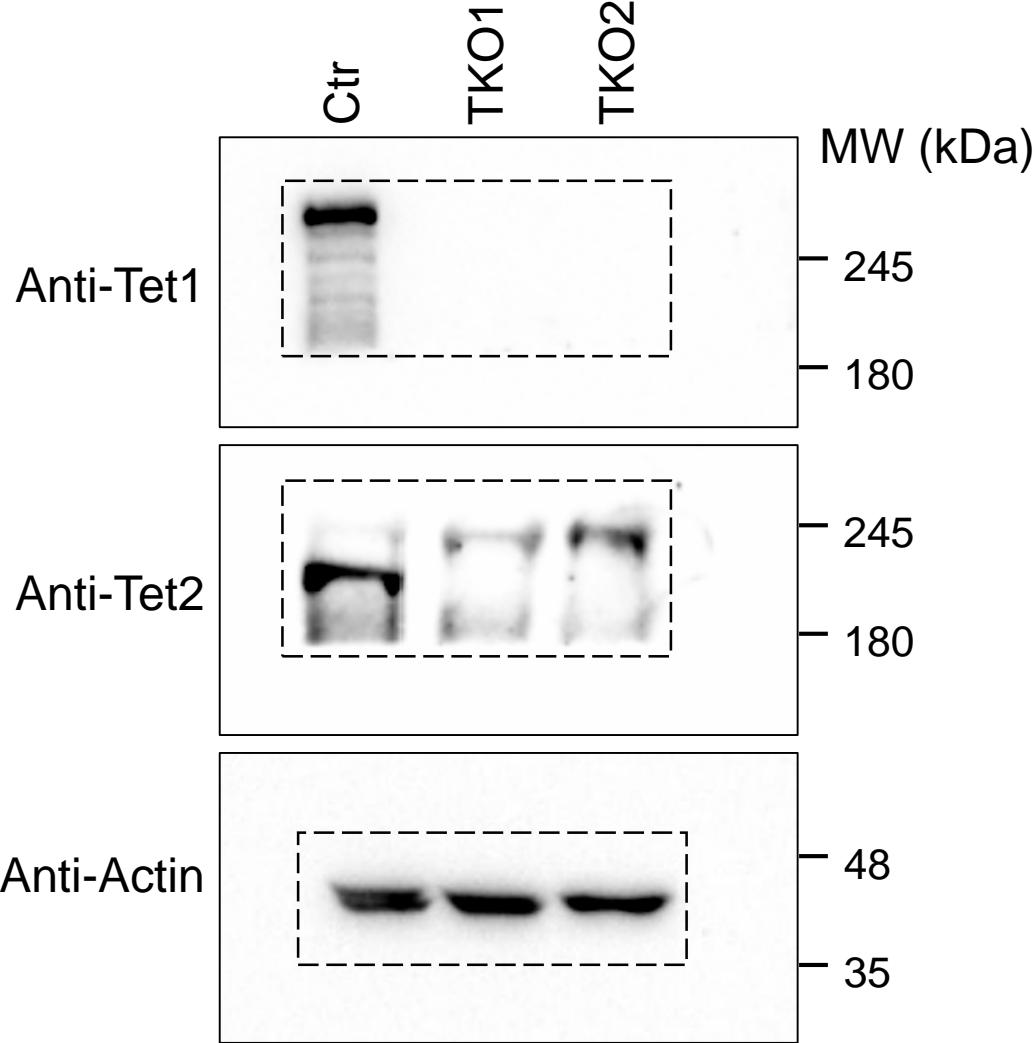

Figure S9H

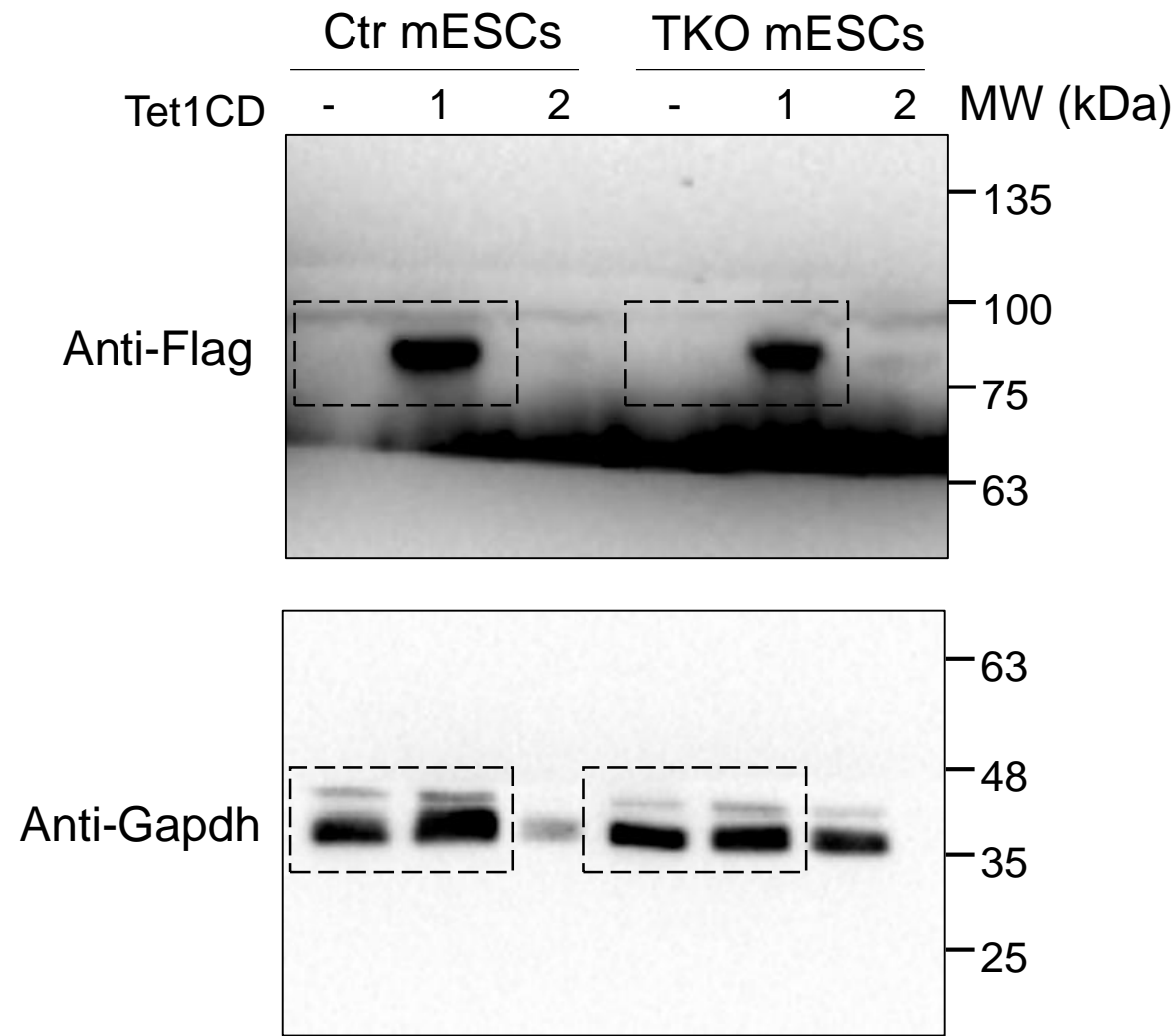

Figure S9I

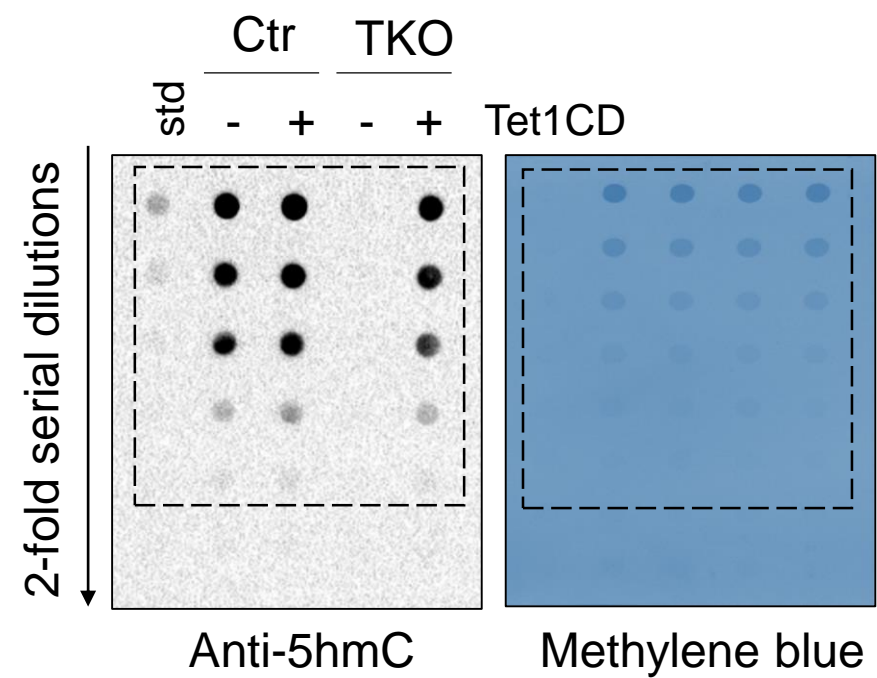

Supplement: Supplementary file 8 — Source Data [file 41467_2019_12325_MOESM8_ESM.zip › Source Data/Source Data_blots.pdf]
